# Supplementary material for: Bacterial load slopes represent biomarkers of tuberculosis therapy success, failure, and relapse
Source: Commun Biol. 2021 Jun 2;4:664. doi: 10.1038/s42003-021-02184-0 (PMC8172544; doi:10.1038/s42003-021-02184-0)
Supplement: Supplementary file 2 — Supplementary Information [file 42003_2021_2184_MOESM2_ESM.pdf]

Bacterial load slopes represent biomarkers of tuberculosis therapy success, failure, and relapse

Gesham Magombedze, PhD[1], Jotam G. Pasipanodya, MD[1,2], Tawanda Gumbo, MD[1, 2]

[1]Center for Infectious Diseases Research and Experimental Therapeutics (CIDRET), Baylor Research Institute, Dallas, Texas, USA; [2]Quantitative Preclinical and Clinical Sciences Department, Praedicare Inc, Dallas, Texas, USA.

Correspondence: E-mail:gmagombedze@gmail.com

Supplementary Material

Table S1. Estimated kill rates and initial bacterial load by cluster versus period of data derivation.

Table S2. Cluster summary for bacterial burden

Table S3. Two month-derived γ_s magnitude cut-off values and initial bacteria burden

Table S4. Contingency tables of outcomes versus different biomarkers

Table S5: Converting time to positivity to colony forming units per milliliter.

Table S6: Growth parameters for bacterial subpopulations

Figure S1. Converting TTP to CFU and vise-versa

Figure S2: Model fitting to accrued data at 8-weeks, 4-month and 6-month time points

Figure S3: Evolution of treatment outcome biomarkers.

Figure S4. Biomarker characterization in indeterminate data zones.

Figure S5: Thresholds for regions with mixed outcomes

Table S1: Estimated kill rates and initial bacterial load by cluster versus period of data derivation.

Period in which data was derived	Cluster	$\log_{10} B_f(0)$	f	γ_f	γ_s
**First 8-weeks**	**Cure**	5.18 (4.91-5.45)	0.66 (0.61-0.73)	0.80 (0.62-0.98)	0.17 (0.16-0.19)
	**Slow cure**	5.10 (4.78-5.37)	0.71 (0.64-0.79)	0.75 (0.54-0.97)	0.17 (0.15-0.19)
	**Relapse**	5.02 (4.57-5.50)	0.81 (0.66-0.89)	0.75 (0.36-0.99)	0.15 (0.11-0.18)
	**Failure**	5.35 (4.78-5.86)	0.65 (0.60-0.81)	0.60 (0.41-0.93)	0.12 (0.09-0.15)
**First 4-months**	**Cure**	4.87 (4.59-5.15)	0.61 (0.60-0.63)	0.60 (0.48-0.80)	0.13 (0.12-0.15)
	**Slow cure**	4.79 (4.52-5.10)	0.61 (0.60-0.64)	0.45 (0·40-0·66)	0.12 (0.11-0.13)
	**Relapse**	4.87 (4.43-5.43)	0.67 (0.60-0.80)	0.39 (0.30-0.91)	0.11 (0.10-0.13)
	**Failure**	5.30 (4.71-5.81)	0.64 (0.60-0.75)	0.57 (0.40-0.90)	0.11 (0.10-0.12)
**6 Months**	**Cure**	4.57 (4.33-4.84)	0.60 (0.60-0.62)	0.50 (0.42-0.65)	0.12 (0.11-0.13)
	**Slow cure**	4.46 (4.25-4.68)	0.60 (0.60-0.62)	0.41 (0.36-0.49)	0.11 (0.10-0.11)
	**Relapse**	4.78 (4.39-5.27)	0.63 (0.60-0.73)	0.36 (0.30-0.72)	0.10 (0.10-0.11)
	**Failure**	5.10 (4.56-5.56)	0.62 (0.60-0.70)	0.51 (0.36-0.80)	0.09 (0.09-0.10)

Estimates for kill rate are given as median values obtained from the model to data fitting of the different patient treatment outcome clusters. The uncertainties in the estimates are given by 95% credible intervals. $B_f(0)$ in the initial log phase bacterial load and the semi-dormant bacteria, $B_f(0)$, is estimated as $f \times B_f(0)$, where f estimates the fraction of $B_s(0)$ with respect to $B_f(0)$. The kill rates were estimated with the 2, 4 and 6 months-derived data sets. The prior of log10 $B_f(0)$ = 4- 6, f=0.5-1, $\gamma_f = 0.5 - 1.0$, γ_s=*0-0.5*.

Table S2. Cluster summary for bacterial burden

Cluster	6-Months (log10 CFU/mL)	9-Months (log10 CFU/mL)	12-Months (log10CFU/mL)	15-Months (log10 CFU/mL)	18-Months (log10 CFU/mL)
**Cure**	0.24	0.24	0.24	0.24	0.24
**Slow cure**	0.24 (0.24-0.40)	0.24 (0.24-0.43)	0.24 (0.24-0.40)	0.24 (0.24-0.39)	0.24 (0.24-0.48)
**Relapse**	0.24 (0.24-1.11)	2.20 (0.31-4.40)	1.65 (0.24-4.43)	2.10 (0.24-3.88)	0.24 (0.24-1.00)
**Failure**	3.19 (1.42-4·46)	0.66 (0.24-1.00)	0.73 (0.24-2.53)	0.64 (0.24-1.84)	2.61 (1.06-4.50)

Summary estimates for different clusters during follow up period. Median estimates are given and the 1st and 3rd quartile values are given in brackets. The value 0.24 log10 CFU represent the limit of detection that corresponds to 42 days for TTP MGIT readouts.

Table S3. Two month-derived γ_s magnitude cut-off values and initial bacteria burden

**Conditional cure regions**	**γ_s cut-off [log_{10} CFU/mL/day]**	**γ_s cut-off [TTP day]**	**Initial bacterial [TTP/day]**
6 months outcomes	$0.1<\gamma_s<0.15$	$-3.9<\gamma_s<-2.6$	-<8.11
4 months outcomes	$0.09<\gamma_s<0.14$	$-2.86<\gamma_s<-2.34$	-<5.93
**Failure regions**			
6 months outcomes	<0.1	< -2.60	-
4 months outcomes	<0.1	< -2.60	-
**High chance of relapse regions**			
4 months and 6 months outcomes	$0.1<\gamma_s<0.15$	$-3.9<\gamma_s<-2.6$	<-5.49

Table S4. Contingency tables of outcomes versus different biomarkers

	8week-Biomarker		Extended EBA		2 month smears	
	**Positive**	**Negative**	**Positive**	**Negative**	**Positive**	**Negative**
**6 months therapy duration**						
Cure	3	155	30	155	20	117
Failure	34	26	5	13	10	47
**4 months therapy duration- isoniazid arm**						
Cure	13	361	53	344	36	299
Failure	56	55	6	15	20	47
**4 months therapy duration- ethambutol arm**						
Cure	30	338	77	378	61	334
Failure	69	137	9	23	25	76

Table S5: **Converting time to positivity to colony forming units per milliliter**.

**Model**	**α**	**β**	**γ**	**AIC**	**BIC**	**R^2**
**Combined log-phase growth and slow/non-replicating *Mtb***						
$F = \alpha e^{-\beta x}$	8.19(8.04,8.34)	0.084(0.08,0.088)	-	-1945	-1936	0.94
$F = \alpha e^{-\beta x+\gamma}$	8.09(6.64,9.96)	0.084(0.08,0.087)	0.011(-0.2,0.2)	-1939	-1925	0.94
**Log-phase growth data**						
$F = \alpha e^{-\beta x}$	8.50(8.39,8.62)	0.081(0.078,0.084)	-	-1768	-1760	0.98
$F = \alpha e^{-\beta x+\gamma}$	9.44(8.08,9.98)	0.081(0.078,0.084)	-0.103(-0.163,0.047)	-1762	-1749	0.98
**Semidormant/non-replicating persisters data**						
$F = \alpha e^{-\beta x}$	7.25(6.82,7.61)	0.096(0.086,0.106)	-	-358	-352	0.96
$F = \alpha e^{-\beta x+\gamma}$	7.84(6.08,9.99)	0.095(0.083,0.104)	-0.081(-0.331,0.159)	-352	-343	0.96

Estimated parameters for converting TTP values to CFU values. Uncertainty of the parameter estimates are given by 95% credible intervals in brackets. AIC and BIC values show how fitting the model with 2 parameters (α and β) compares to fitting with 3 parameters (α, β and γ). R^2 is the goodness of fit.

Table S6: Growth parameters for bacterial subpopulations

**Parameters**	r_f **(per day)**	r_s **(per day)**	K_{max} **x 10**^{8} **(constant)**
Priors	0.0-1.0	0.0-1.0	0.5
Estimates	0.1775 (0.07,0.36)	0.0787 (0.029,0.099)	Fixed

Growth rates and the maximum bacterial carrying capacity was estimated using Hollow fiber data with MTB clinical isolates (Magombedze et al., [24]).

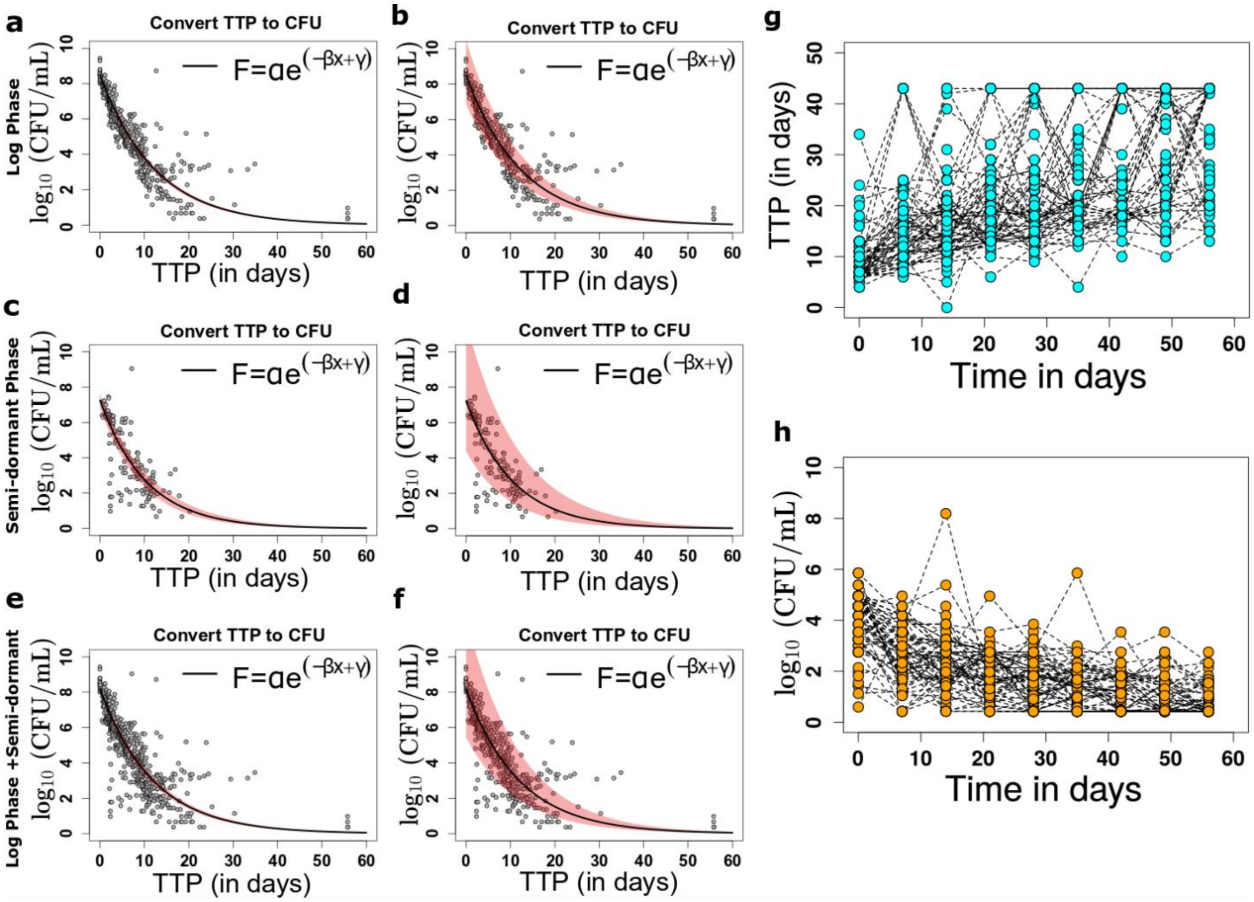

Figure S1. Converting TTP to CFU and vise-versa. Illustrating conversion of serial TTP values from patients to corresponding CFU values. Panels **a** showing model function correlating TTP to CFU, here the model has two parameters to the estimated, in **b** the model with three parameters has wider uncertainty. While conversion for bacteria in log phase is shown in **a** and **b**, conversion for slow and non-replicating bacteria is shown in **c** and **d**, then in panels **e** and **f**, combined subpopulations are shown. Panel **g** shows TTP patient clinical data and the corresponding converted CFU are shown in **h** at start of therapy [Day 0] and every 7 days till day 56.

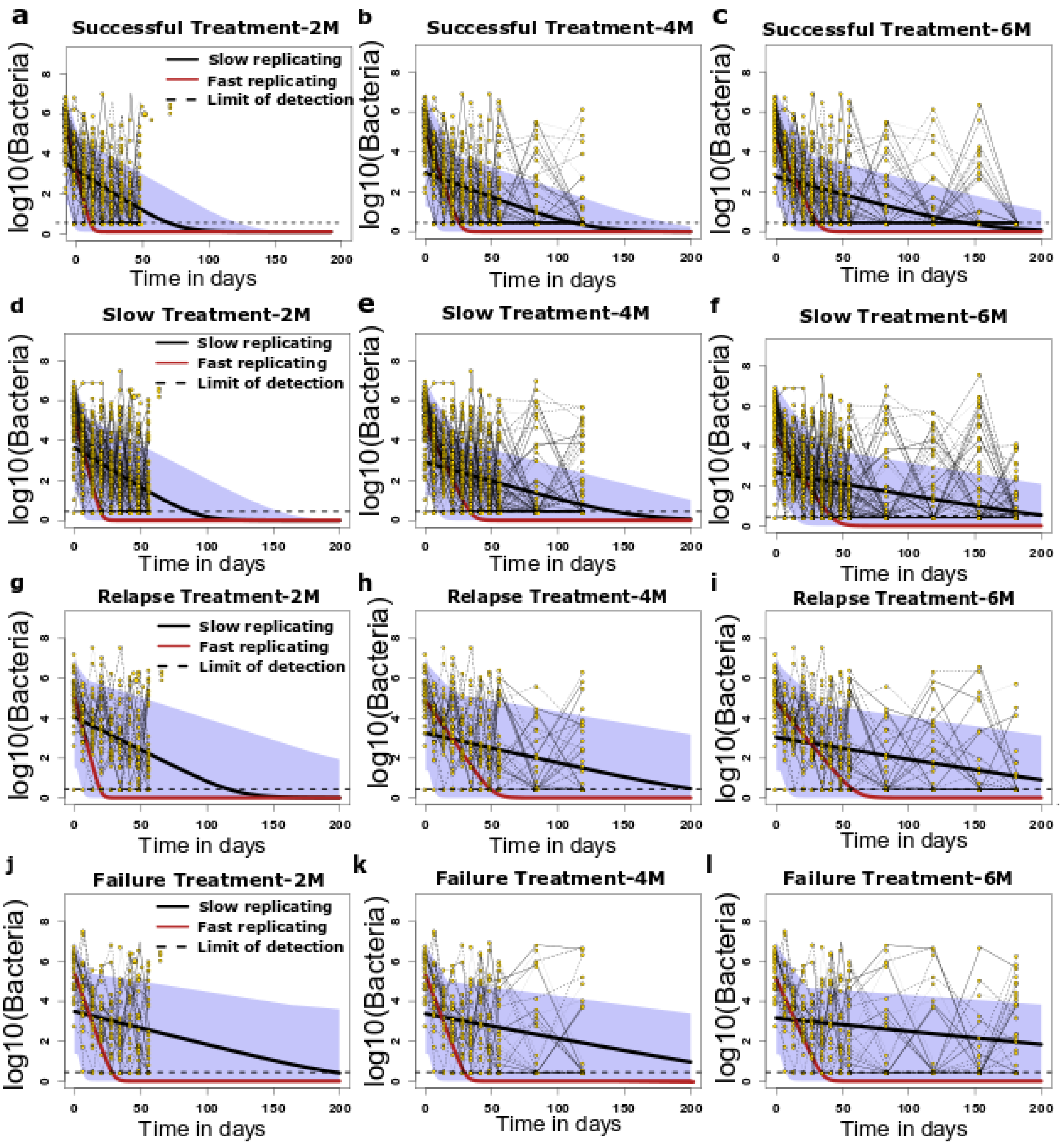

Figure S2. Model fitting to accrued data at 8-weeks, 4-months and 6-months time points. Shown are model fitting to the accrued cure data cluster at 8-weeks/2M (**a**), 4M (**b**) and 6M (**c**), respectively. Model fitting to the slow successful cure cluster data are shown in **d** (at 2M), **e** (at 4M) and **f** (at 6M). Relapsing disease data patterns are given in **g**, **h**, and **i** and while treatment failure are shown in **j**, **k** and **l** at 2M, 4M and 6M, respectively. The gold dots represent the observations, the solid lines are the model predictions and the shaded regions represent the 95% credible intervals.

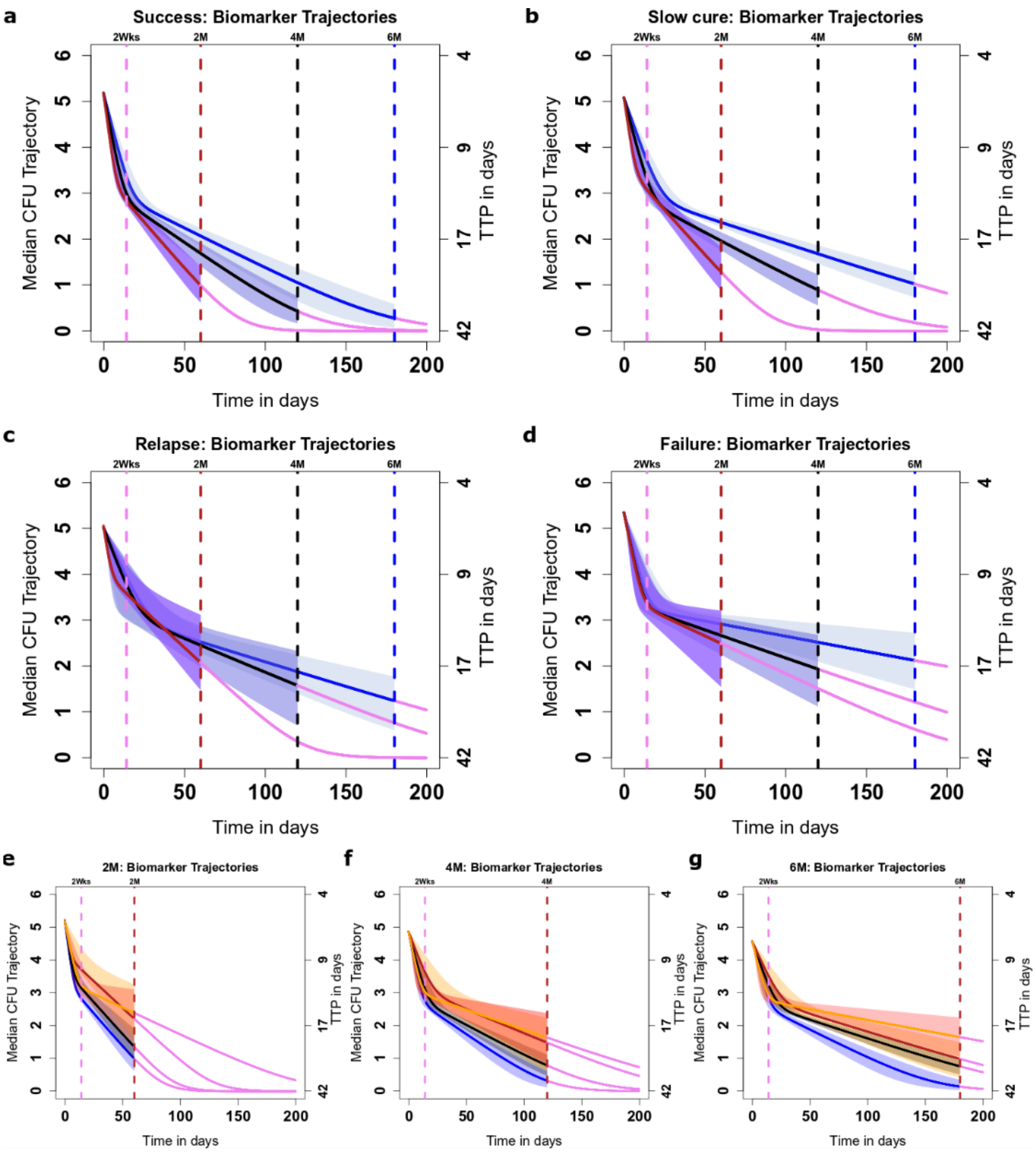

Figure S3: Evolution of treatment outcome biomarkers. (**a**) The evolution of biomarkers patterns that identifies cured patients at 2M**,** 4M and 6M, respectively. Summary trajectories for patients in the slow successful treatment cluster (**b**), relapsing cases (**c**) and treatment failure cluster (**d**) demonstrate how these patterns diverge. Further comparisons of all biomarkers for each cluster and their respective 95% credible intervals at 2M (**e**), at 4M (**f**) and at 6M (**g**) end points are shown.

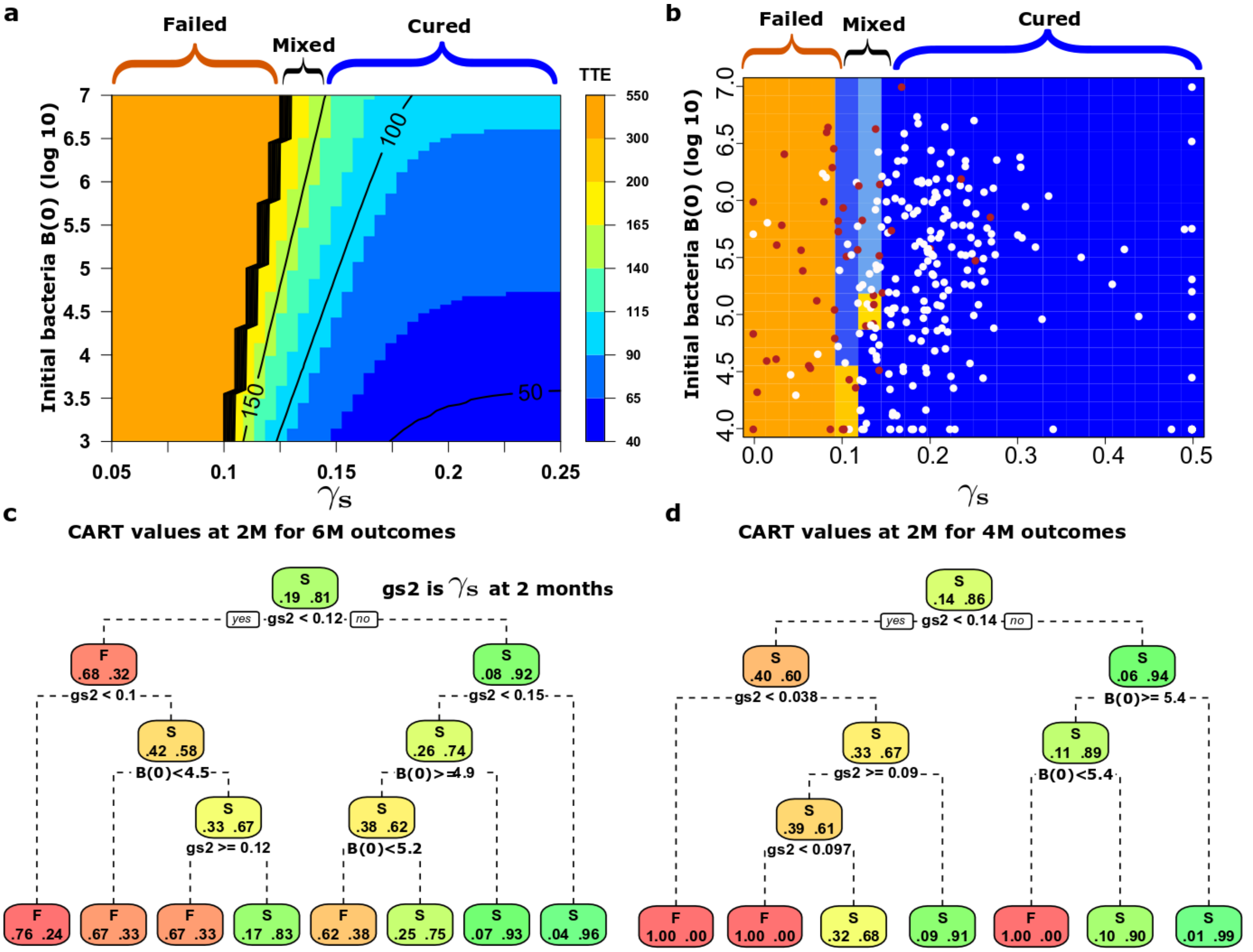

Figure S4. Biomarker characterization in indeterminate data zones. Thresholds for the biomarkers that enable treatment outcome predictions at 2, 4 and 6-months are shown. (**a**) Simulation-based derivation of the biomarker thresholds and how they vary with initial patient bacteria burden, $B(0)$. (**b**) A combination of γ_S-slopes and $B(0)$ in classifying outcomes classification regression trees (CART). In **c** and **d**, shown are the biomarker breakpoints predicted with CART using 6M and 4M treatment outcomes, respectively. In **c**), failures at 6 months are predicted with a γ_S-slope that is less than 0.1. In **d**), the 4-month failure threshold is predicted to be 0.1(0.097), while 0.14 predicts cures. Both in **c** and **d**, the zone between γ_S-slope of 0.1 and 0.15 has high misclassification. The mixed regions in **a** and **b** demonstrate conditional outcomes dependent on initial bacterial burden and γ_S rates (**Figure S4**).

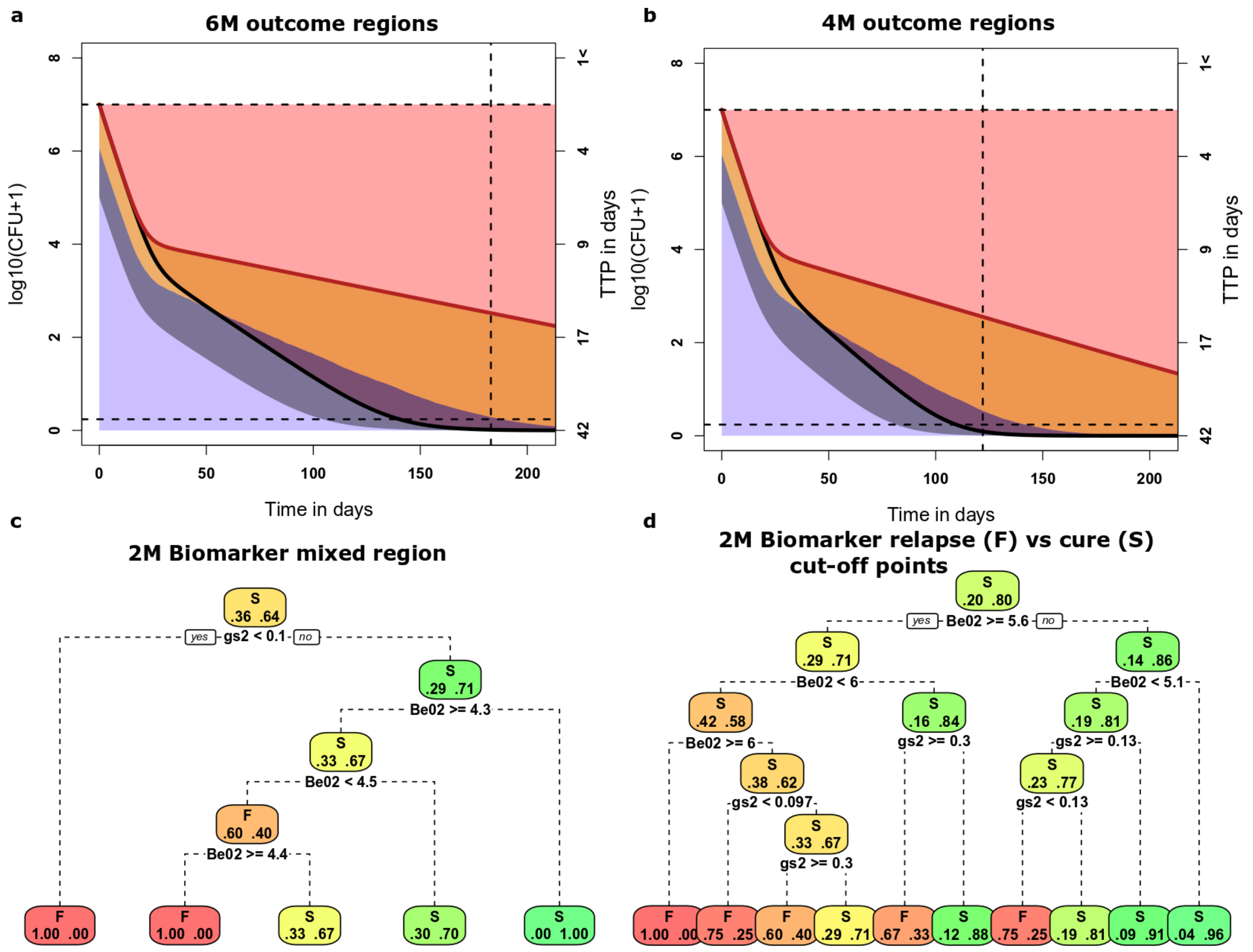

Figure S5. **Thresholds for regions with mixed outcomes**. γ_s values greater than 0.125 cure patients with initial bacteria burden between 4-6 log 10CFU/mL and values below 0.125 will result in failure using simulations. **(b)** illustrates the overlap between the initial bacteria and the slopes for the 4-month outcomes. In **a** and **b** regions of failure are shaded orange and the cure region has the grey shading). (**c)** CART derived cut-off values for 6 months outcomes. (**d**) CART derived predictor (initial burden and γ_s-slope) cut-off to delineate relapses from cure.
